# Targeting p53 in Cancer: Functional States, Therapeutic Strategies, and Clinical Progress

**DOI:** 10.3390/cancers18121861

**Published:** 2026-06-06

**Authors:** Anais Saunders, Joshua Barkin, Anthony Karnezis, Jeremy Chien

**Affiliations:** 1 Department of Biochemistry and Molecular Biology, University of California, Davis, CA 95817, USA; asaunders@health.ucdavis.edu (A.S.); jbarkin@health.ucdavis.edu (J.B.); 2 Department of Pathology and Laboratory Medicine, University of California, Davis, CA 95817, USA; ankarnezis@health.ucdavis.edu; 3 Department of Obstetrics and Gynecology, University of California, Davis, CA 95817, USA

**Keywords:** p53-targeted therapies, reactivation, targeted-degradation, neoantigen-driven adoptive cell therapies

## Abstract

*TP53* is the most frequently altered tumor-suppressor gene in human cancer, yet efforts to therapeutically target p53 have yielded limited and inconsistent clinical success. We argue that this gap reflects not a lack of druggable biology, but an oversimplified conceptual framework that treats p53 as a binary wild-type versus mutant entity. Here, we synthesize emerging evidence supporting a model in which p53 operates across a spectrum of functional states defined by mutation class, allelic burden, isoform composition, aggregation propensity, post-translational regulation, and cellular context. These states shape distinct biological outputs, including transcriptional activity, dominant-negative and gain-of-function effects, immune modulation, and checkpoint dependency, which collectively determine therapeutic vulnerability. We review current strategies targeting the p53 pathway, including mutant p53 reactivation, targeted degradation, anti-aggregation approaches, immune-directed therapies, restoration of wild-type pathway activity, gene replacement, and synthetic lethal targeting of DNA damage response dependencies. Clinical and preclinical evidence highlights key limitations of each approach, including stoichiometric constraints, mutation specificity, context-dependent efficacy, and adaptive resistance. Notably, emerging evidence from preclinical and correlative clinical studies suggests that therapeutic outcomes may be more closely associated with p53 functional state than with *TP53* mutation status alone. We further emphasize the emerging roles of p53 isoforms and the tumor immune microenvironment as critical modifiers of p53 activity and determinants of treatment response. Collectively, these insights support a paradigm shift toward mechanism-matched, biomarker-stratified strategies that align therapeutic modality with the operative p53 network. Future progress will depend on integrating multi-parameter diagnostics with rational combination therapies to fully exploit p53 as a central vulnerability in cancer.

## Simple Summary

This review summarizes current therapeutic strategies targeting p53, including approaches to reactivate mutant p53, promote its degradation, prevent protein aggregation, exploit synthetic lethal vulnerabilities, and engage immune-based therapies, and highlights why many of these strategies have shown limited success when applied broadly. Rather than viewing p53 as simply wild-type or mutant, we refocus on a framework in which p53 dysfunction is understood as a spectrum of clinically relevant functional states shaped by mutation type, protein behavior, isoform expression, and tumor context. These distinct states influence tumor biology and therapeutic response, helping to explain inconsistent clinical outcomes across p53-targeted therapies. By aligning specific treatment strategies with defined p53 functional states, this framework may support a more precise, biomarker-driven approach to targeting the p53 pathway and improving therapeutic efficacy in cancer.

## 1. Introduction

*TP53* is among the most intensively studied tumor-suppressor genes and is now understood as a master regulator of cellular stress responses and cell fate decisions. Although originally misclassified as an oncogene due to early studies examining mutant alleles, *TP53* was later redefined as a tumor suppressor when these transforming activities were traced to missense mutations [1,2]. Wild-type (WT) p53 protein levels are tightly controlled by a negative feedback loop involving the E3 ubiquitin ligase MDM2. Cellular stressors including DNA damage, oncogene activation, metabolic stress, and chemotherapy stabilize p53 and activate transcriptional programs governing cell-cycle arrest, DNA repair, apoptosis, ferroptosis, metabolism, senescence, immune regulation, and metastasis inhibition [1].

*TP53* is the most frequently altered tumor-suppressor gene in human cancer, with mutations present in ~50% of all malignancies and exceeding 90% in cancer types such as high-grade serous ovarian carcinoma (HGSOC), small cell lung cancer (SCLC), head and neck squamous cell carcinoma (HNSCC), and triple-negative breast cancer (TNBC) [3,4]. Over 70% of these alterations are missense mutations within the DNA-binding domain (DBD), generating proteins that can be transcriptionally incompetent, dominant-negative over residual WT p53, and/or oncogenic gain-of-function (GOF) drivers [3,4]. The *TP53* locus encodes at least twelve p53 isoforms, generated through alternative promoter usage, splicing, and translation initiation. These isoforms frequently modulate p53 activity independently of coding mutations, and through dominant-negative or neomorphic effects can render tumors with wild-type *TP53* functionally p53-deficient [5,6].

Considering its central role in tumor suppression and frequent mutations and pathway alterations in human cancer, the *TP53* gene and its product p53 have been a target of therapeutic development to restore tumor-suppressive function of mutant p53. Despite decades of intensive effort, most p53-targeted therapies have failed to deliver durable clinical benefit. We argue that these failures reflect not only limitations in drug design, including insufficient potency and off-target effects, but also flawed biological stratification, treating p53 as a binary variable rather than a dynamic, state-dependent network.

**Figure 1:**
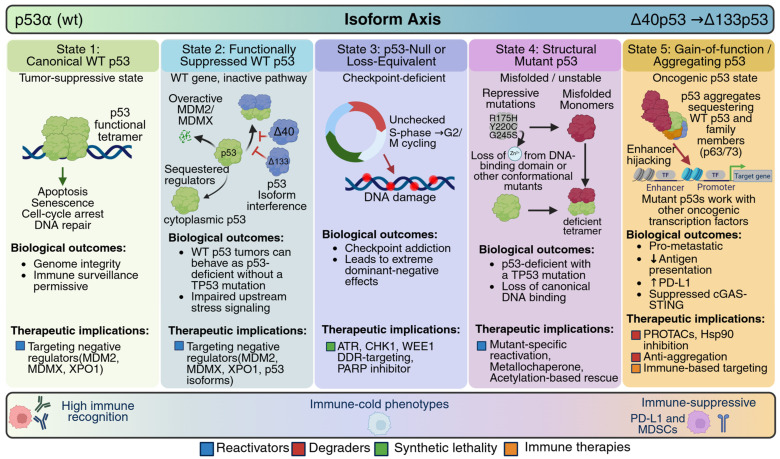
Functional states of the p53 network in cancer and their therapeutic implications. Schematic representation of p53 as a spectrum of functional states rather than a binary wild-type versus mutant entity. p53 output is shaped by mutation class (contact vs. conformational), allelic dosage and dominance, isoform composition (e.g., full-length p53α, Δ40p53, and Δ133p53), post-translational modification, protein stability, aggregation behavior, and cellular context. These states determine distinct biological consequences, including altered transcriptional programs, aggregation-mediated dominant-negative and gain-of-function effects, immune suppression, and checkpoint dependency. Clinically, these states can be approximated using integrated genomic, proteomic, and transcriptomic features. Therapeutic strategies shown include mutation-selective reactivation, zinc metallochaperone rescue, targeted degradation, anti-aggregation approaches, immune-directed therapies (vaccines and engineered TCR-T cells), synthetic lethal targeting of checkpoint addiction, and pathway reactivation in wild-type but suppressed p53. The figure emphasizes that optimal therapeutic benefit requires mechanism-matched, state-aware intervention rather than reliance on *TP53* mutation status alone. Created in BioRender. Barkin, J. (2026) https://BioRender.com/6aivca3.

New treatment paradigms conceptualize p53 not as a binary WT–mutant variable, but as a spectrum of ‘functional states’ integrating output of mutation class, allelic dominance, isoform expression, aggregation behavior, pathway suppression, and immune context (Figure 1). In this framework, a ‘p53 functional state’ is defined as an integrated biological condition reflecting the net output of the p53 network, determined by mutation class (e.g., contact vs. conformational), allelic status, isoform composition, protein stability and aggregation behavior, post-translational regulation, and tumor microenvironmental context. Distinct states are distinguished by measurable features such as transcriptional competency, dominant-negative or gain-of-function activity, aggregation propensity, immune-modulatory signaling, and checkpoint dependency. Clinically, assignment to a functional state can be approximated using a combination of next-generation sequencing (mutation type and allelic fraction), protein-level assays (IHC and aggregation markers), transcriptomic profiling (p53 target gene signatures and isoform expression), and immune biomarkers (e.g., PD-L1 and MHC expression). This operational definition is intended to guide biomarker-driven stratification and mechanism-matched therapeutic selection.

Therapeutic success will be determined by alignment between drug mechanism and this composite state. This expanded view helps reconcile decades of inconsistent therapeutic outcomes, in which *TP53* mutation status alone proved insufficient to predict response to p53-targeted agents [1,7]. In this new treatment paradigm, trial design, therapeutic mechanism, and biomarker selection are matched to the operative p53 network state-based p53 biology rather than to mutation status alone.

## 2. p53 Biology and Pathophysiology

The p53 protein comprises N terminal transactivation domains, a proline-rich region, a central DNA-binding domain, a tetramerization domain, and a C terminal regulatory tail. Tetrameric p53 binds sequence specific DNA response elements to regulate canonical targets such as CDKN1A (p21), BAX, and PUMA, integrating stress signals into arrest, repair, or death programs [1]. Missense *TP53* mutations are highly enriched in the DBD and are commonly categorized as conformational (structural) mutants, which destabilize folding (e.g., R175H and Y220C), or contact mutants, which preserve global folding but disrupt DNA interactions (e.g., R248Q/W and R273H) [3].

Beyond loss of tumor-suppressor activity, many p53 mutants exert dominant-negative effects on WT p53 tetramers and acquire GOF properties that include enhancer hijacking, chromatin remodeling, altered metabolism, invasion, metastasis, and immune modulation [4,7]. These oncogenic activities are highly context-dependent, varying with tissue lineage, cooperating mutations, and microenvironmental cues.

**Figure 2:**
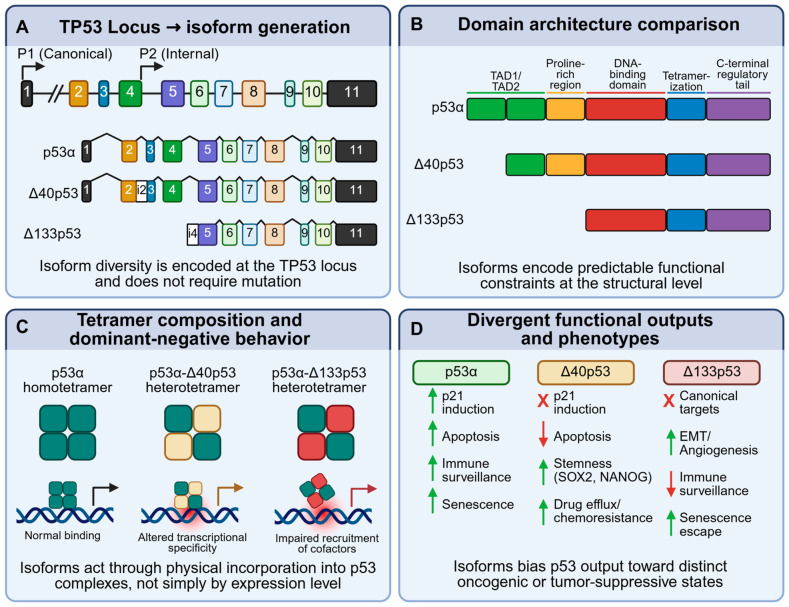
p53 isoforms define distinct functional states and act as predictive biomarkers in cancer. (**A**) Schematic of the *TP53* locus illustrating generation of multiple p53 isoforms through alternative promoter usage (P1 and P2), splicing, and translation initiation, producing proteins with distinct N- and C-terminal truncations (e.g., p53α, Δ40p53, and Δ133p53 isoforms). (**B**) Functional consequences of major isoforms. Δ40p53 retains partial transactivation capacity but selectively impairs induction of key targets such as CDKN1A, uncoupling apoptosis from cell-cycle arrest and promoting stemness, invasion, and chemoresistance. In contrast, Δ133p53 isoforms lack canonical transactivation domains and drive non-canonical transcriptional programs associated with epithelial–mesenchymal transition, angiogenesis, immune suppression, and escape from senescence. (**C**) Isoform-driven modulation of p53 functional states. Isoforms hetero-oligomerize with full-length and mutant p53, altering tetramer composition, transcriptional output, and pathway activity independent of *TP53* mutation status. (**D**) Different isoforms produce divergent transcriptional outputs, tumor cell phenotypes, and functional outcomes. The relative abundance and ratios of p53 isoforms define a distinct functional state that influences tumor phenotype, therapeutic sensitivity, and clinical outcome, positioning isoform profiles as emerging biomarkers for patient stratification and response prediction. Created in BioRender. Barkin, J. (2026) https://BioRender.com/e8jog5v.

In addition to somatic mutations, the biological output of p53 signaling is also profoundly shaped by isoform expression (Figure 2). Δ40p53 retains partial transactivation capacity yet selectively fails to induce CDKN1A, uncoupling apoptosis from cell-cycle arrest and promoting stem-like phenotypes, chemoresistance, and invasion [6,8]. In contrast, Δ133p53 isoforms lack canonical transactivation domains but drive non-canonical transcriptional programs linking p53 dysregulation to epithelial–mesenchymal transition, angiogenesis, and immune suppression [5,9].

In parallel, many structural p53 mutants undergo amyloid-like aggregation, forming oligomeric assemblies that sequester wild-type p53, p63, and p73, thereby amplifying dominant-negative and gain-of-function effects [10,11]. Aggregation thus represents a biophysical failure mode of the p53 network and defines a therapeutically actionable state distinct from transcriptional incompetence alone.

## 3. Mutant p53 and the Tumor Immune Microenvironment

**Figure 3:**
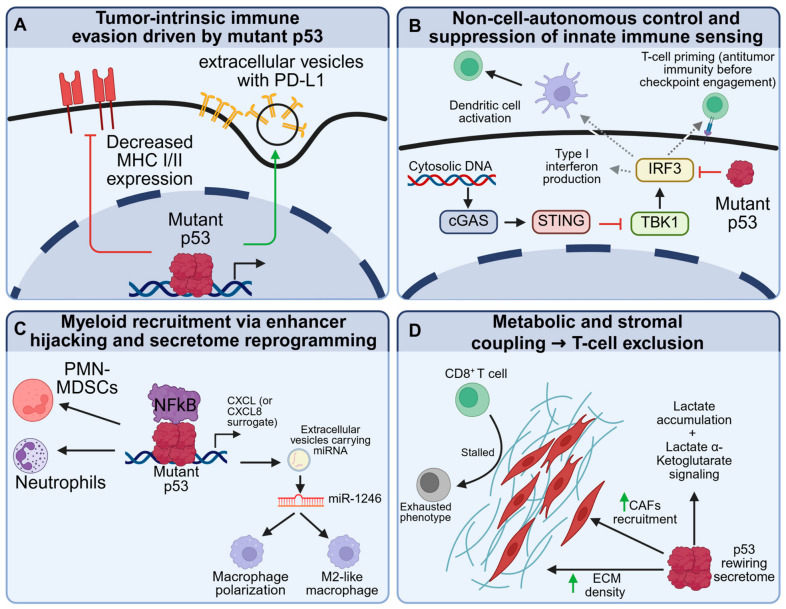
Mutant p53 shapes an immunosuppressive tumor microenvironment as a distinct p53 functional state. (**A**,**B**) Tumor-intrinsic immune evasion mechanisms. Gain-of-function mutant p53 suppresses antigen presentation via downregulation of MHC class I/II; p53 loss increases immune checkpoint ligands such as PD-L1 and inhibits innate immune sensing through repression of the cGAS–STING–TBK1–IRF3 axis, limiting T-cell priming and effector activation. (**C**) Remodeling of the tumor immune microenvironment. Mutant p53 cooperates with inflammatory transcriptional programs (e.g., NF-κB) and enhancer hijacking to induce chemokines and cytokines that recruit immunosuppressive populations, including M2-like macrophages, myeloid-derived suppressor cells, and neutrophils, while excluding cytotoxic CD8^+^ T cells. (**D**) Non-cell-autonomous and metabolic reprogramming. Mutant-p53-dependent secretory factors, extracellular vesicles, and metabolic byproducts (e.g., lactate) reinforce immune suppression and stromal remodeling. Collectively, these mechanisms define an immunosuppressive p53 functional state that constrains responsiveness to immunotherapy and provides a rationale for immune-directed strategies, including checkpoint blockade combinations and TCR-engineered cell therapies targeting mutant p53 neoantigens. Created in BioRender. Barkin, J. (2026) https://BioRender.com/qvr9aqn.

Mutant p53 is increasingly recognized as a central architect of an immunosuppressive tumor microenvironment, exerting effects across adaptive immunity, innate immunity, stromal compartments, and tumor cell-intrinsic signaling [12,13,14,15,16] (Figure 3). *TP53* mutations promote deficiencies in antigen-presentation machinery (MHC I/II), increase PD-L1 expression through relieving miRNA-mediated repression [17,18,19] and through other pathways [20,21], and suppress innate immune sensing through the inhibition of the cGAS–STING–TBK1–IRF3 axis [22], collectively reducing T-cell priming and activation. Chronic inflammatory signaling driven by GOF p53 mutants promotes T-cell exhaustion and immune escape.

Beyond tumor-intrinsic effects, mutant p53 reprograms stromal and immune compartments through enhancer hijacking and metabolic rewiring. GOF mutants bias macrophage polarization toward M2-like phenotypes, alter cancer-associated fibroblast behavior, and reshape extracellular-matrix composition, while metabolic byproducts such as lactate and α-ketoglutarate further modulate chromatin state and immune-gene expression [14]. Mechanistically, certain p53 mutants hijack enhancers to activate chemokines such as CXCL1 in cooperation with NF-κB, selectively recruiting polymorphonuclear myeloid-derived suppressor cells (MDSCs) and neutrophils while excluding cytotoxic CD8^+^ T cells; genetic ablation of *Cxcl1* reverses these immunosuppressive phenotypes in vivo [15]. In parallel, mutant-p53-dependent secretome and extracellular-vesicle reprogramming, including export of miRNAs such as miR-1246, propagates immunosuppressive polarization across the tumor niche.

In hematologic malignancies, *TP53* mutations are associated with impaired antigen presentation, expansion of Tregs and MDSCs, NK-cell dysfunction, and poor responses to allogeneic hematopoietic stem cell transplantation and immunotherapy, reinforcing mutant p53 as a determinant of immune resistance across tumor classes [16]. Gain-of-function p53 mutants actively sculpt an immunosuppressive tumor microenvironment through both tumor-intrinsic and non-cell-autonomous mechanisms. Mutant p53 represses antigen presentation via downregulation of MHC I/II, promotes immune checkpoint engagement through PD-L1 upregulation, and suppresses innate immune sensing by inhibiting the cGAS–STING–TBK1–IRF3 axis [12,22]. These immune-evasive programs provide a mechanistic explanation for the limited efficacy of p53 vaccines as monotherapies and motivate direct antigen-based strategies that bypass endogenous priming.

## 4. Therapeutic Strategies Targeting Mutant p53

### 4.1. p53 Gene-Replacement Strategies

Gene-replacement approaches represent the earliest clinical attempts to restore p53 function. Adenoviral delivery of wild-type p53 (Ad5CMV-p53, rAd-p53, commercially known as Gendicine) demonstrated safety and biological activity but rarely produced durable regressions as monotherapy in Western trials [23,24]. In contrast, Gendicine has been used clinically in China for head and neck cancer for over two decades, often in combination with radiotherapy [25].

Second-generation delivery platforms such as SGT-53, a transferrin-targeted p53 nanocomplex, improve tumor uptake and exhibit synergy with chemotherapy and immune checkpoint inhibitors in preclinical models of pancreatic, lung, and breast cancer [26,27]. These advances support renewed interest in p53 gene therapy as part of combination regimens rather than standalone treatment.

### 4.2. Reactivation of Mutant p53

Reactivation strategies aim to restore WT-like conformation and transcriptional activity to mutant p53. Allele-specific, small-molecule stabilization of the Y220C mutant exploits a mutation-induced surface pocket which has been targeted since the late 2000s with compounds such as PhiKan083. PhiKan083 was identified as a Y220C stabilizer using in silico screening, and later as potentially rescuing the Y220S mutant too [28,29]. It was the precursor to additional small molecules such as PC14586, also known as Rezetapopt, and demonstrates strict genotype dependence in preclinical and early clinical studies [30]. Unlike solid tumors, leukemias have a relatively higher frequency of Y220C p53 mutations, making them good candidates for treatment with PC14586. This highlights the feasibility of precision p53 rescue for defined patient subsets. Recent efforts to target the Y220C mutant by Coultreon Biopharma have yielded a group of small-molecule candidates that promise improved cellular potency and sustained activity compared to PC14586 [31].

Although mutant p53 reactivation remains conceptually appealing, its clinical realization is constrained by fundamental stoichiometric and mechanistic barriers. Many p53 mutants oligomerize with residual mutant subunits, such that partial conformational rescue may be functionally silent in the presence of dominant-negative tetramers [7]. Consequently, effective rescue requires near-complete engagement of the mutant pool at clinically achievable drug exposures. These challenges are exemplified by APR-246 (eprenetapopt), whose cytotoxicity is now appreciated to arise largely from p53-independent mechanisms rather than from systematic transcriptional reactivation of mutant p53 [32,33]. APR-246, also known as PRIMA-1(Met), is the derivative of PRIMA-1 and is converted intracellularly to methylene quinuclidinone (MQ). MQ depletes glutathione, perturbs redox homeostasis, induces ROS accumulation and ferroptotic cell death, and sensitizes tumors to chemotherapy regardless of *TP53* status [34,35,36]. Consistently, therapeutic response to APR-246 correlates more strongly with redox markers such as SLC7A11/xCT expression than with structural rescue of mutant p53 [32].

A mechanistically distinct class comprises zinc metallochaperones such as ZMC1, which restore Zn^2+^ binding and stabilize zinc-deficient conformational mutants, particularly R175H. ZMC1 can also bind Cu^2+^, which unlike Zn^2+^, is a redox-active metal that induces the production of ROS [37,38,39]. Interestingly, ROS production was found to be a key factor for the stabilization and restoration of p53 transcriptional activity upon ZMC1 treatment [39]. Under wild-type p53 conditions, cellular stress signals, such as that of oxidative stress, promotes post-translational modification (PTM) of p53, which stabilizes it, protects it from degradation by MDM2, and allows it to become transcriptionally active. Functional rescue by ZMC1 is stress-dependent and coupled to post-translational modifications that activate downstream p53 transcriptional programs [39,40]. Collectively, reactivation strategies succeed only when mutant p53 abundance, aggregation state, and redox vulnerability align; and these factors must be prospectively assessed and stratified to improve the success of reactivation strategies in future clinical trials.

### 4.3. Degradation of Mutant p53

Targeted degradation of mutant p53 provides an orthogonal strategy that circumvents the stoichiometric constraints of conformational rescue and may be a preferred approach in mutant p53 biologic states dominated by gain-of-function effects, immune suppression, aggregation and dominant-negative effects. By selectively eliminating mutant proteins, degraders neutralize dominant-negative and neomorphic activities without relying on transcriptional reactivation [7]. Peptide- and aptamer-based proteolysis-targeting chimeras (PROTACs) targeting hotspot alleles such as R175H demonstrate allele-specific degradation, inhibition of invasion and metastasis, and tumor regression in preclinical models [41,42]. However, degradation strategies do not restore canonical p53 tumor-suppressor function, underscoring a central trade-off between eliminating oncogenic activity and reinstating physiologic stress responses.

Early efforts focused on Hsp90 inhibitors, such as onalespib and ganetespib, exploiting the dependence of misfolded p53 mutants on chaperone support. Although these agents reduced mutant p53 levels and suppressed tumor growth in preclinical models [43,44], clinical performance was limited and therapeutic effects cannot be assigned solely to p53. In ovarian cancer, the phase 2 GANNET53 trial demonstrated that combining ganetespib with paclitaxel failed to improve progression-free survival and increased serious adverse events, underscoring the disconnect between biological rationale and therapeutic index for indirect degraders [45].

More recently, mutation-selective degradation strategies have emerged. Peptide-based PROTACs and RNA or DNA aptamer-guided PROTACs have been designed to selectively target hotspot mutants, most notably p53 R175H, inducing proteasome-dependent degradation while sparing wild-type p53 [41,42]. These agents suppress migration, invasion, and clonogenic growth in vitro and reduce tumor burden in xenograft models, establishing proof-of-principle for allele-restricted degradation. However, their translation is limited by delivery challenges, mutant specificity that constrains patient coverage, and the conceptual limitation that degradation mitigates GOF phenotypes without restoring canonical p53 tumor-suppressor function [7].

### 4.4. Anti-Aggregation Strategies

**Figure 4:**
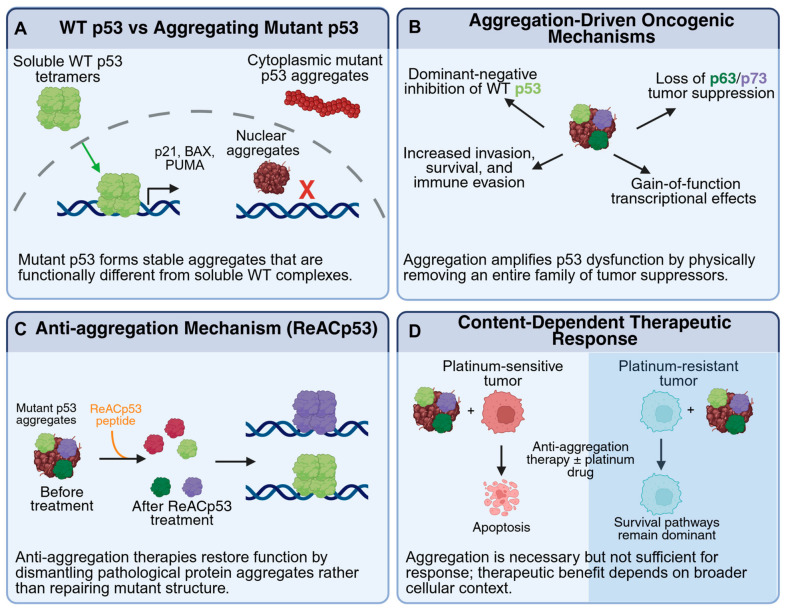
Anti-aggregation therapy targets an aggregation-dominant p53 functional state. (**A**) Structural basis of aggregation. Destabilizing *TP53* mutations promote misfolding of the DNA-binding domain and formation of amyloid-like oligomers and aggregates. (**B**) Dominant-negative and gain-of-function consequences. Aggregated mutant p53 sequesters wild-type p53 and its paralogs p63 and p73, amplifying loss-of-function and oncogenic gain-of-function activities. (**C**) Mechanism of anti-aggregation therapy. The cell-penetrant peptide ReACp53 binds aggregation-prone motifs, prevents oligomer formation, and shifts mutant p53 toward a soluble conformation. (**D**) Functional restoration and therapeutic implications. Dissolution or prevention of aggregates releases sequestered p53 family proteins, enabling reactivation of tumor-suppressive transcriptional programs, including apoptosis. Therapeutic efficacy depends on mutation class, aggregation propensity, residual wild-type protein, and treatment context, defining aggregation as a distinct and targetable p53 functional state requiring mechanism-matched intervention. Created in BioRender. Barkin, J. (2026) https://BioRender.com/elayjg0.

Anti-aggregation therapies target a fundamental biophysical pathology of mutant p53. A growing body of evidence indicates that many structural p53 mutants form amyloid-like aggregates that sequester wild-type p53, p63, and p73, exacerbating loss-of-function and GOF effects [11,46]. Anti-aggregation strategies aim to dissociate or prevent these assemblies and thereby restore transcriptional competence (Figure 4).

The cell-penetrant peptide ReACp53 binds aggregation-prone motifs within the DNA-binding domain, shifting mutant p53 toward a soluble, transcriptionally competent state while preventing sequestration of wild-type family members [11]. ReACp53 restores p53 target gene expression, induces apoptosis, and suppresses tumor growth in models of high-grade serous ovarian and prostate cancer [11,47]. Importantly, synergy with carboplatin has been observed predominantly in platinum-sensitive tumors, whereas platinum-resistant and p53-null contexts derive limited benefit, highlighting the dependence of therapeutic efficacy on mutation class and treatment history [11,48].

Additional aggregation-modulating mechanisms include regulation by the chaperone/disaggregase DAXX, bifunctional ligands that combine metallochaperone and anti-aggregation activity, and the emerging recognition that RNAs can influence mutant-p53 phase behavior and solubility. DAXX is a protein known to be involved in the regulation of p53 and MDM2 [49,50,51]. DAXX has been characterized as a “disaggregase” and an “unfoldase” [52]. It is a polyD/E protein that has been shown to prevent aggregation of R175H and R280K mutant p53 [52]. This study suggests that upregulating DAXX expression in the context of mutant p53 could reduce aggregation and restore cell death activation by p53. However, it is important to note that this is highly context-dependent. Indeed, knockdown of DAXX in the context of the wild-type p53-expressing cell line SK-N-SH results in an increased induction of apoptosis [49]. While knockdown may be beneficial for cells harboring wild-type p53, overexpression may provide restorative effects for mutant p53-expressing cells that undergo the formation of aggregates. Other approaches include the use of bifunctional ligands, L^I^ L^H^, that act to both prevent aggregation and act as metallochaperones, as we previously discussed, to stabilize mutant p53 [53,54]. Additionally, there is evidence to suggest that the conversion of mutant p53 from a functional soluble form to an aggregate form is mediated by RNAs [55]. This offers another set of targets for the modulation of p53 aggregates. Together, these findings support aggregation as both a pathogenic mechanism and a druggable vulnerability in a subset of p53-mutant cancers.

### 4.5. Synthetic Lethality in TP53-Deficient Tumors

**Figure 5:**
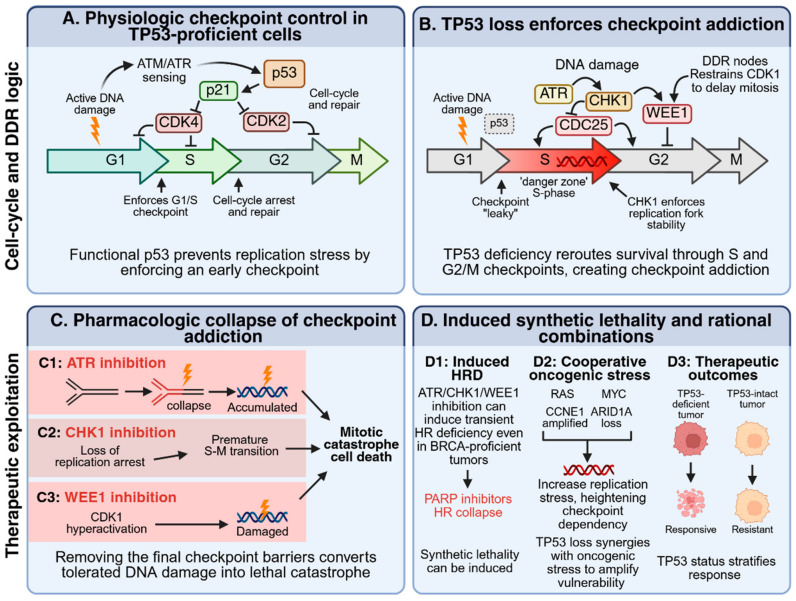
Loss of p53 abolishes the G1/S checkpoint, forcing *TP53*-deficient tumors to rely on S-phase and G2/M DNA damage checkpoints for survival. (**A**) Loss of p53 function abrogates the G1/S checkpoint, primarily through loss of CDKN1A (p21)–mediated inhibition of CDK2, forcing cancer cells to rely on S-phase and G2/M checkpoints to maintain genomic integrity under replicative stress. (**B**) This dependency creates a state of checkpoint addiction, rendering TP53-deficient tumors selectively vulnerable to disruption of the ATR–CHK1–WEE1 signaling axis. (**C**) Pharmacologic inhibition of ATR, CHK1, or WEE1 in TP53-deficient cells prevents proper DNA damage resolution, resulting in unscheduled mitotic entry with unresolved DNA lesions, and ultimately mitotic catastrophe and cell death. (**D**) This vulnerability is amplified by oncogenic replication stress (e.g., MYC or RAS activation, CCNE1 amplification) and can be further exploited through induced homologous recombination deficiency and rational combination therapies. Created in BioRender. Barkin, J. (2026) https://BioRender.com/kz8tyfj.

Loss of p53 disables the G1/S checkpoint by eliminating p21-mediated CDK inhibition and enforces reliance on S-phase and G2/M checkpoints, creating a state of checkpoint addiction that can be therapeutically exploited (Figure 5). S- and G2/M-phase checkpoints are governed by ATR, CHK1, and WEE1, and the pharmacologic inhibition of this axis results in replication catastrophe and mitotic failure in *TP53*-deficient cells.

Clinical validation is strongest for WEE1 inhibition with adavosertib, which improved progression-free survival in *TP53*/RAS-mutant colorectal cancer and other high-replication-stress tumors [56]. ATR inhibitors similarly demonstrate enriched activity in *TP53*- and ATM-deficient contexts and can induce a transient homologous recombination deficiency, sensitizing tumors to PARP inhibitors [57,58,59]. Co-alterations such as CCNE1 amplification, MYC overexpression, and ARID1A loss further heighten checkpoint addiction and refine patient selection [58,60,61].

Recent work has demonstrated that distinct classes of mutant p53 proteins differentially regulate DNA replication dynamics and tumor immunogenicity, with important implications for therapy. In a study by Liu et al., contact mutations such as p53 R273H were shown to override normal replication checkpoint control by promoting persistent TopBP1–Treslin interactions, leading to excessive replication initiation and genomic instability [62]. This replication “over-firing” results in increased formation of micronuclei and release of cytosolic DNA, which activates the cGAS–STING innate immune pathway and induces anti-tumor immune responses. In contrast, conformational mutants such as p53 R175H promote tumor growth without triggering comparable replication stress or immune activation, underscoring mutation-specific functional divergence. Importantly, tumors harboring contact mutant p53 exhibited increased immune cell infiltration and enhanced responsiveness to immune checkpoint inhibition in preclinical models, linking replication stress to immunogenicity. These findings define a replication stress-driven, immune-activated p53 functional state and support the concept that specific *TP53* mutations can predict response to immunotherapy and expose vulnerabilities to replication-targeting agents such as ATR or PARP inhibitors.

### 4.6. Immune-Directed Targeting of Mutant p53

Because mutant p53 is expressed at high levels and is tumor-restricted, it represents an attractive immunologic target. Vaccine-based approaches, including p53MVA, synthetic long peptide (SLPp53) vaccines, and adenovirus-transduced dendritic cell vaccines, reliably induce p53-specific T-cell responses but have shown limited clinical activity as monotherapies [63,64]. Clinical benefit improves when vaccines are combined with immune checkpoint inhibitors, particularly PD1 blockade, and when patients exhibit preserved peripheral immune competence [65].

**Figure 6:**
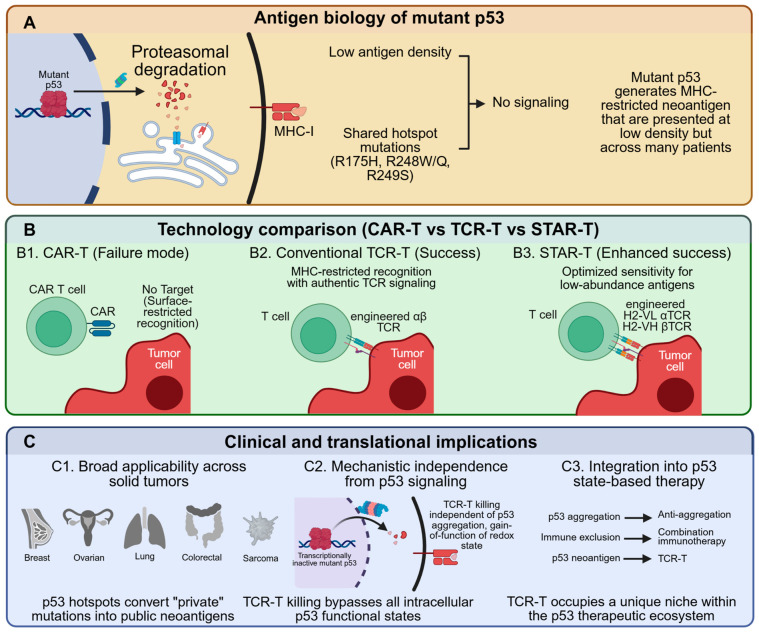
Immune-directed targeting of mutant p53 as a distinct p53 functional state-matched strategy. (**A**) Neoantigens derived from mutant p53 presented at low MHC-I density, leading to ineffective immune response. (**B1**) Mutant p53-driven immunosuppressive functional states, characterized by reduced antigen presentation, checkpoint upregulation, and impaired innate immune signaling, constrain the magnitude and durability of CAR-T responses. (**B2**) Engineered T cell receptor (TCR) therapies targeting shared p53 neoantigens. TCR-engineered T cells recognize intracellular mutant p53 peptides presented on MHC class I molecules with high sensitivity, enabling the targeting of low-abundance antigens that are inaccessible to antibody-based approaches. (**B3**) Synthetic TCR and Antigen Receptor (STAR) T cells are developed to enhance sensitivity for low-abundance intracellular neoantigens. (**C**) Clinical and conceptual implications. Immune-directed therapies bypass the need for biochemical restoration of p53 function and instead exploit mutant p53 as a tumor-restricted antigen. Therapeutic efficacy depends on HLA genotype, mutation-specific epitope presentation, and immune competence, defining an immunogenic p53 functional state that can be selectively targeted using mechanism-matched immunotherapies. Created in BioRender. Barkin, J. (2026) https://BioRender.com/ua42pt1.

Adoptive T-cell therapies targeting mutant p53 neoantigens are emerging as a promising new modality for treating solid tumors that harbor recurrent *TP53* hotspot mutations. Unlike p53-reactivating drugs or traditional immune therapies, these approaches exploit the fact that many *TP53* missense mutations produce shared, tumor-specific neoantigens that can be targeted across patients, especially when paired with common HLA alleles (Figure 6). Engineered T cell receptor (TCR) therapies targeting shared p53 neoantigens represent the most mechanistically orthogonal p53-directed strategy to date.

Early foundational work [66] demonstrated that *TP53* hotspot mutations generate immunogenic neoantigens across diverse epithelial cancers, with 18% of screened patients showing T-cell responses. Importantly, the immunogenicity extended across multiple hotspot mutations, including R175H, R248W/Q, and R249S, indicating that these recurrent mutations create a broad therapeutic landscape rather than isolated targets. The study also found that immunogenicity depended largely on mutant peptide-HLA structural features, establishing the feasibility of building a library of mutant-specific TCRs covering a substantial fraction of cancer patients [66,67].

Building on this foundation, the Rosenberg group from NCI provided the first clinical proof-of-concept that engineered TCR-T cells can target these shared p53 neoantigens [68]. They analyzed 163 metastatic cancer patients, identifying 39 TCR recognizing *TP53* hotspot mutations, thus validating the concept of “public neoantigen” [69]. They reported a landmark case: a woman with metastatic breast cancer harboring p53 R175H achieved 55% tumor regression lasting six months after receiving engineered peripheral T cells expressing an HLA-A*02:01-restricted anti-R175H TCR. This was the first demonstration that an off-the-shelf, mutation-specific TCR can produce meaningful tumor regression in a solid tumor. Mechanistically, the relapse in this patient was highly informative: the recurrent lesion exhibited loss of heterozygosity at HLA-A*02:01, providing that HLA loss is a direct resistance mechanism to TCR-T cell therapy.

This insight shaped the next wave of product engineering, where an armored R175H-specific TCR-T cell therapy incorporated (1) CRISPR knockout of endogenous TCRα/β (preventing mispairing), (2) knock-in of the high-affinity anti-R175H TCR, and (3) disruption of TGFBR2 to eliminate TGF-β-mediated immunosuppression. The first dedicated phase 1 trial building on this concept is NT-175, reported in preliminary form in a conference abstract [70], and therefore represents early-stage clinical evidence. This trial enrolls HLA-A*02:01 patients with solid tumors harboring R175H, across multiple cancer types, highlight the pan-tumor potential of *TP53*-hotspot-directed TCR therapy.

Conventional CAR-T cells are 10–100-fold less sensitive than TCR-T cells when targeting HLA class I-presented neoantigens at low density, because CARs lack the CD3 signaling complex and co-receptor components that amplify TCR signal transduction. To close this gap, Huang et al. engineered a Synthetic TCR and Antigen Receptor (STAR) [71], a chimeric construct that pairs a TCR-mimicking single-chain variable fragment (scFv, derived from the H2 TCR-mimic antibody targeting the p53R175H/HLA-A*02:01 complex) with the intracellular signaling components of the endogenous TCR-CD3 complex rather than the CD28/4-1BB domains used by CARs. This enables STAR-T cells to activate via the full TCR signaling cascade upon neoantigen engagement. Preclinical studies suggest that STAR-T cells may exhibit improved sensitivity compared to CAR-T and TRuC-T (TCR fusion construct) platforms in low-neoantigen-density settings and better tumor control; however, these findings are currently limited to in vitro and xenograft models and require validation in immunocompetent and clinical contexts. The study directly addresses a fundamental limitation of applying established CAR-T technology to MHC-I-restricted intracellular neoantigens like mutant p53. Because p53 peptides are presented at low MHC-I density, authentic TCR signaling, rather than CAR-based recognition, is required for sufficient sensitivity. These STAR-T and TCR replacement platforms represent a viable approach to mutation-specific immunotherapy [71].

### 4.7. Mechanism-Specific Resistance and Adaptive Responses

Therapeutic resistance across p53-targeted modalities reveals mechanistically distinct but conceptually convergent escape pathways that are consistent with a functional-state model of p53 biology. For MDM2 inhibitors, resistance is frequently driven by genetic selection, with multiple studies demonstrating the emergence and clonal expansion of *TP53*-mutant cells during treatment, effectively converting tumors from a wild-type p53-dependent state into a functionally p53-deficient state that is intrinsically resistant to further pathway activation [72]. In contrast, resistance to XPO1 inhibitors such as selinexor is largely mediated by adaptive signaling rewiring, including activation of ATR–CHK1-dependent DNA damage response pathways, NF-κB-driven survival signaling, and upregulation of XPO1 itself, which collectively restore tumor cell viability and limit sustained nuclear p53 activity [73,74]. Similarly, resistance to p53 reactivators such as APR-246 involves both pathway-level adaptation and network-level suppression, including activation of TGF-β–THBS1 signaling loops and increased nuclear export that attenuate effective p53 transcriptional reprogramming [73,75].

Recent studies have significantly revised the mechanistic basis of response to the p53-reactivating drug APR-246 (eprenetapopt), demonstrating that sensitivity is primarily determined by cellular redox state rather than *TP53* mutation status alone. In particular, the expression of the cystine/glutamate antiporter SLC7A11 (xCT), which regulates cystine uptake and glutathione biosynthesis, has emerged as a dominant predictor of response, outperforming *TP53* mutation status across large cancer cell line panels [32]. High xCT expression promotes glutathione production and enhances cellular antioxidant capacity, thereby buffering the oxidative stress induced by APR-246 and conferring resistance. Conversely, low xCT expression results in reduced glutathione availability and increased susceptibility to oxidative damage. Mechanistically, APR-246 depletes glutathione and induces lipid peroxidation, triggering ferroptotic cell death in a manner that is partially independent of p53 reactivation [34]. This process critically involves glutathione peroxidase 4 (GPX4), the central enzyme that detoxifies lipid hydroperoxides using glutathione as a cofactor; impairment of the xCT–glutathione–GPX4 axis therefore sensitizes tumors to ferroptosis. Consistent with this model, inhibition of SLC7A11 or GPX4 synergizes with APR-246 to enhance tumor cell killing. Although the extent to which the xCT-GSH-GPX4 axis plays a role in resistance to APR-246 in clinical setting has yet to be defined, it warrants further investigation.

Collectively, these findings illustrate that resistance to p53-targeted therapies does not arise from a single mechanism but rather depends on the drug mechanisms and p53 functional states, including genetic loss of p53 activity, adaptive checkpoint dependence, and suppression of p53 signaling output. This reinforces the need for mechanism-matched combination strategies that anticipate functional states and target both primary vulnerabilities and adaptive escape pathways.

## 5. Therapeutic Strategies Targeting the Wild-Type p53 Pathway

In tumors retaining wild-type *TP53* but exhibiting pathway suppression, therapeutic efforts focus on releasing negative regulation or enforcing nuclear function. MDM2 inhibitor Nutlin-3 was discovered in the early 2000s and is still considered the gold-standard positive control for confirming wild-type-like activity in the laboratory. However, due to poor PK/PD, it required re-engineering of the chemical structure to yield clinically viable candidates. These efforts resulted in compounds such as idasanutlin and milademetan, which stabilize p53 and induce cell-cycle arrest; however, their effects are frequently cytostatic rather than cytotoxic, and resistance commonly arises through *TP53* mutation, BCL-XL upregulation, or adaptive pathway rewiring [76,77,78,79,80].

Dual inhibition of MDM2 and MDMX offer broader pathway release. Agents such as ALRN-6924 and APG-115 show enhanced activity in wild-type p53 settings and synergize with BCL-2 inhibitors or immune checkpoint blockade in preclinical models [81,82,83]. Clinical development has increasingly emphasized combination strategies and biomarker-defined populations.

P53 proteostasis extends beyond classic MDM2 feedback loops to incorporate proteins involved in transcription-coupled repair and cellular stress recovery. Specifically, CSA and CSB proteins, frequently mutated in Cockayne syndrome, assemble with p53, MDM2, DDB1, and Cullin-4 ubiquitin ligase complexes to accelerate MDM2-dependent p53 degradation [84]. Depletion of CSA or CSB prevents efficient p53 ubiquitination, inducing sustained p53 stabilization, elevated apoptotic cascades, and chronic stress signaling independent of *TP53* mutation status. Ultimately, these observations demonstrate that functional p53 signaling states can be driven by altered protein turnover and disrupted stress-adaptive networks rather than primary gene mutations alone.

Inhibition of nuclear export via XPO1 blockade (Selinexor) promotes nuclear retention of p53 and has demonstrated clinical benefit in wild-type-p53 endometrial cancer, with subgroup analyses revealing strong p53-status dependence [85,86]. Trials in other malignancies, including DLBCL, now explicitly incorporate p53 status into eligibility criteria, reflecting a maturation toward state-based trial design.

## 6. p53 Isoforms as Biomarkers and Therapeutic Targets

Emerging evidence indicates that distinct p53 isoforms exert functionally divergent and clinically relevant effects that help define tumor-specific p53 states beyond *TP53* mutation status alone. The canonical full-length p53α isoform remains the primary mediator of tumor-suppressive activity, driving transcriptional programs that induce cell-cycle arrest, apoptosis, and DNA repair; however, its activity is rarely isolated and is modulated by co-expressed isoforms, consistent with the concept that cellular responses reflect the integrated output of the p53 isoform network [87]. Complementary integrated genomic studies further demonstrate that variability in isoform expression, linked to *TP53* sequence context and co-altered pathways, is associated with tumor behavior and clinical progression, supporting their role as independent or additive biomarkers [88,89].

Among alternative isoforms, p53γ retains partial transcriptional competency and has been associated with improved clinical outcomes, particularly in *TP53*-mutant breast cancers where p53γ expression can partially restore tumor-suppressive signaling and improve prognosis relative to mutant p53 alone [90]. In contrast, truncated isoforms generated by internal promoter usage exhibit distinct oncogenic properties. The Δ133p53 isoforms (Δp53α/β/γ) lack the first transactivation domain but retain partial regulatory function, and have been shown to promote tumor progression, inflammation, immune evasion, and metastasis while exerting dominant-negative effects on full-length p53. Clinically, elevated Δ133p53β is associated with poor prognosis and metastatic progression across multiple cancer types [91,92], particularly in tumors retaining wild-type *TP53* [89]. Mechanistically, Δ133 isoforms engage in non-canonical transcriptional programs linked to invasion, epithelial–mesenchymal transition, and microenvironment modulation [91,92]. In comparison, the Δ160p53 isoforms (Δ160p53α/β/γ) are even more truncated, lacking both transactivation domains and part of the DNA-binding region, and display more pronounced GOF oncogenic activity characterized by enhanced proliferation, stemness, and resistance to apoptosis, along with stronger interference with p53α activity [89,92].

This progressive truncation from Δ133 to Δ160 defines a spectrum of increasing functional divergence from tumor-suppressive to oncogenic behavior. Functionally, these isoforms map onto distinct therapeutic vulnerabilities: tumors dominated by p53α-functional states may retain sensitivity to MDM2 inhibitors and DNA-damaging therapies; γ-enriched states may exhibit enhanced apoptotic priming and improved therapeutic responsiveness. Tumors expressing β isoforms linked to poor prognosis in otherwise *TP53* wild-type settings may benefit from interventions that enhance canonical p53 activity or disrupt dominant-negative isoform interactions. Inhibition of NMD rescues p53β/γ isoform expression and reactivates p53 signaling in MDM2-driven p53-deficient tumors [93]. Mechanistically, β and γ isoforms are less susceptible to MDM2-mediated degradation [93], and they can amplify residual p53 activities especially in wild-type or partial functional contexts [94]. Similar to aggregation-prone missense mutants, the Δ133p53β isoform has higher propensity for aggregation [95] and a dominant-negative effect on wild-type p53 [94]. Interestingly, the p53 peptide (derived from truncated p53 sequence 107–129) can prevent the aggregation of Δ133p53β [95]; thus, tumors with a high expression of Δ133p53β isoforms may be responsive to anti-aggregation strategies.

Δ133-driven states may also favor immune-modulatory or anti-metastatic strategies; whereas Δ160-dominant states, reflecting a more severe loss of canonical p53 activity and gain of oncogenic functions, may require alternative approaches such as targeting downstream signaling dependencies, ferroptosis-based therapies, or synthetic lethal vulnerabilities. Δ40p53-dominant states associated with impaired apoptosis and enhanced survival signaling may predict resistance to DNA-damaging chemotherapy and favor combination strategies that restore apoptotic competence.

These results indicate that p53 isoform expression patterns and ratios may, in some contexts, provide additional predictive and theranostic value beyond canonical mutation-based stratification. Collectively, they underscore the clinical importance of systematically measuring p53 isoform expression in tumor samples because such profiling has the potential to improve prognostic accuracy, explain discordance between *TP53* mutation status and clinical outcomes, and enable more precise, mechanism-matched stratification of patients for targeted therapies.

## 7. Clinical Lessons and Future Directions

Various therapeutic classes, representative agents and approaches, target p53 states, primary mechanisms, key biomarkers for selection, clinical status and key lessons are outlined in Table 1.

Three principles emerge from three decades of p53-targeted therapy development. First, monotherapies are rarely sufficient; durable benefit typically requires rational combinations. Second, *TP53* mutation status alone is inadequate. Allelic burden, zygosity, isoform expression, aggregation state, immune contexture, and co-occurring genomic alterations critically shape response. Third, the therapeutic mechanism must be matched to p53 functional state: reactivation faces stoichiometric and dominant-negative barriers, whereas degradation neutralizes GOF but does not restore tumor suppression [7].

Collectively, these advances argue against universal p53 reactivation paradigms and instead support mechanism-matched, biomarker-stratified strategies tailored to defined p53 functional states. Durable benefit is most likely to arise from combinatorial approaches that integrate mutant-specific targeting, immune modulation, synthetic lethality, and isoform biology. Future trials must therefore evolve from mutation-based enrollment toward multi-parameter stratification frameworks that reflect the full complexity of the p53 regulatory network [4,7].

Recent computational and functional studies identify TGF-β–THBS1 signaling that blunts responses to p53 reactivator APR-246, and perturbation of these pathways restores sensitivity [75]. Another study by Wang et al. reported that APR-246 activates multiple cell death mechanisms independent of p53 [33]. Our recent studies indicate that off-target effects of APR-246 on ferroptosis often mask the on-target effect on rescuable mutant p53 such that ferroptosis-resistant cells may be needed to observe the on-target effect of APR-246 [96]. These studies highlight the potential challenges of off-target therapeutic effects that may act independently of p53. Future progress will depend on integrating mutation-specific therapies, differentiating on-target therapeutic effects from off-target toxicity, synthetic lethal strategies, precision immunotherapy, isoform-aware biomarkers, and advanced delivery platforms into state-based, biomarker-stratified clinical trials.

## 8. Limitations

This review is subject to several limitations. First, much of the supporting evidence for the functional state framework derives from preclinical models and retrospective or correlative clinical studies rather than prospective biomarker-stratified trials. Second, the proposed classification of p53 functional states remains conceptual and requires standardized assays and validation across diverse tumor types. Third, many therapeutic strategies discussed exhibit context-dependent efficacy and may not be broadly generalizable. Finally, the complexity of the p53 network, including isoforms, post-translational regulation, and microenvironmental influences, presents ongoing challenges for clinical implementation and a limitation in clinical feasibility of p53 functional states as diagnostics.

Translation of the functional state framework into clinical practice will require robust and accessible diagnostic platforms. Currently, *TP53* mutation status is routinely assessed using next-generation sequencing, while p53 protein levels can be approximated by immunohistochemistry. Emerging approaches include RNA sequencing for isoform profiling, gene expression signatures to measure transcriptional competency, and proteomic assays to assess post-translational modifications and aggregation. Immune context can be evaluated through PD-L1 staining, MHC expression, and tumor-infiltrating lymphocyte profiling. However, comprehensive functional state classification will likely require integrated multi-omics platforms, which are not yet standardized in routine pathology workflows. The development of scalable, clinically validated assays remains a critical prerequisite for implementing state-based therapeutic stratification.

## 9. Conclusions

Targeting p53 in cancer has long been a compelling yet elusive goal, with decades of effort yielding important biological insights but limited and inconsistent clinical success. This review highlights that these challenges stem not from a lack of druggable opportunities, but from an oversimplified view of p53 as a binary entity. Instead, p53 functions across a spectrum of dynamic states defined by mutation class, isoform composition, protein stability, aggregation behavior, and tumor context, each with distinct biological and therapeutic implications. Recognition of these functional state-dependent differences provides a unifying framework to interpret past failures and to rationally guide future therapeutic development. Moving forward, effective targeting of the p53 pathway must depend on mechanism-matched strategies, biomarker-driven patient stratification, and combination therapies that address both tumor-intrinsic and microenvironmental factors. Integrating mechanism-based diagnostics with emerging modalities, including selective reactivation, degradation, immune targeting, and synthetic lethality, offers a path toward realizing p53 as a central and actionable vulnerability in cancer.

## Figures and Tables

**Table 1:** Summary of therapeutic strategies targeting p53 pathway alterations.

Therapeutic Class	Representative Agents/Approaches	Target p53 Functional State (s)	Primary Mechanism	Key Biomarkers for Selection	Clinical Status/Key Lessons
Mutation-selective reactivation	PC14586 (Y220C)	Structural mutants (Y220C)	Stabilizes mutant conformation via pocket binding	*TP53* Y220C mutation; allelic burden	Most specific rescue strategy; limited to small patient subsets
Pan-reactivation/redox modulation	APR-246; PK11007	Structural and contact mutants (context-dependent)	Covalent/redox effects; ROS induction; partial refolding	Redox markers (xCT/SLC7A11); stress sensitivity	Efficacy often p53-independent; best in combinations
Metallo-chaperone-based rescue	ZMC1; Zn^2+^ supplementation	Zinc-deficient structural mutants (e.g., R175H)	Restores Zn^2+^ binding and folding	Mutation class; zinc affinity; ROS tolerance	Strong preclinical data; delivery and selectivity challenges
Acetylation-targeted rescue	AceTACs (e.g., Y220C-directed)	Select structural mutants	Enhances stabilizing PTMs (p300/CBP)	Mutation class; PTM capacity	Emerging; mutation-restricted; needs in vivo validation
Mutant p53 degradation	Hsp90 inhibitors; PROTACs; aptamer PROTACs	GOF/dominant-negative mutants	Reduces mutant p53 abundance	High mutant p53 protein; GOF transcriptional signatures	Neutralizes oncogenic activity but does not restore WT function
Anti-aggregation strategies	ReACp53; DAXX modulation	Aggregating structural/GOF mutants	Prevents amyloid-like aggregation	Aggregation markers; platinum sensitivity	Context-dependent efficacy; strongest in platinum-sensitive disease
Immune vaccination	p53MVA; SLP-p53; Ad-p53 DC	Mutant p53 (antigen-positive tumors)	Induces p53-specific T cell responses	HLA type; immune competence	Modest monotherapy effects; benefit in combinations
Adoptive T cell therapy	Engineered TCR-T/STAR-T (e.g., R175H)	Shared p53 neoantigens	Direct recognition of mutant p53 peptides	*TP53* mutation + HLA match	Most promising immunologic approach for intracellular p53
WT p53 pathway re-activation	MDM2 inhibitors; MDM2/MDMX inhibitors; XPO1 inhibitors	WT-competent but suppressed p53	Releases negative regulation; nuclear retention	WT *TP53*; pathway suppression; isoforms	Cytostatic effects common; resistance via *TP53* mutation emergence
p53 gene replacement	Ad5CMV-p53; rAd-p53; SGT-53	LOF/null states	Restores WT p53 expression	Delivery efficiency; dominant-negative burden	Limited as monotherapy; renewed interest in combinations
Synthetic lethality (DDR)	WEE1, ATR, CHK1 inhibitors ± PARP inhibitors	p53-null/loss equivalent	Exploits checkpoint addiction	*TP53* LOF; replication	Efficacy requires biomarker-defined selection; combination approaches to overcome adaptive resistance

## Data Availability

No new data were created or analyzed in this study. Data sharing is not applicable to this article.

## Abbreviations

The following abbreviations are used in this manuscript:

GOF	Gain-of-function
LOF	Loss-of-function
STAR	Synthetic TCR and antigen receptor
DBD	DNA-binding domain
HGSOC	High-grade serous ovarian carcinoma
SCLC	Small cell lung cancer
WT	Wild type
HNSCC	Head and neck squamous cell carcinoma
TNBC	Triple-negative breast cancer
PROTAC	Proteolysis-targeting chimera
MDSCs	Myeloid-derived suppressor cells
NK	Natural killer
PTMs	Post-translational modifications
